# Uncultivated Microbial Eukaryotic Diversity: A Method to Link ssu rRNA Gene Sequences with Morphology

**DOI:** 10.1371/journal.pone.0028158

**Published:** 2011-12-08

**Authors:** Marissa B. Hirst, Kelley N. Kita, Scott C. Dawson

**Affiliations:** Department of Microbiology, University of California Davis, Davis, California, United States of America; American Museum of Natural History, United States of America

## Abstract

Protists have traditionally been identified by cultivation and classified taxonomically based on their cellular morphologies and behavior. In the past decade, however, many novel protist taxa have been identified using cultivation independent ssu rRNA sequence surveys. New rRNA “phylotypes” from uncultivated eukaryotes have no connection to the wealth of prior morphological descriptions of protists. To link phylogenetically informative sequences with taxonomically informative morphological descriptions, we demonstrate several methods for combining whole cell rRNA-targeted fluorescent *in situ* hybridization (FISH) with cytoskeletal or organellar immunostaining. Either eukaryote or ciliate-specific ssu rRNA probes were combined with an anti-α-tubulin antibody or phalloidin, a common actin stain, to define cytoskeletal features of uncultivated protists in several environmental samples. The eukaryote ssu rRNA probe was also combined with Mitotracker® or a hydrogenosomal-specific anti-Hsp70 antibody to localize mitochondria and hydrogenosomes, respectively, in uncultivated protists from different environments. Using rRNA probes in combination with immunostaining, we linked ssu rRNA phylotypes with microtubule structure to describe flagellate and ciliate morphology in three diverse environments, and linked *Naegleria* spp. to their amoeboid morphology using actin staining in hay infusion samples. We also linked uncultivated ciliates to morphologically similar *Colpoda*-like ciliates using tubulin immunostaining with a ciliate-specific rRNA probe. Combining rRNA-targeted FISH with cytoskeletal immunostaining or stains targeting specific organelles provides a fast, efficient, high throughput method for linking genetic sequences with morphological features in uncultivated protists. When linked to phylotype, morphological descriptions of protists can both complement and vet the increasing number of sequences from uncultivated protists, including those of novel lineages, identified in diverse environments.

## Introduction

Protists have been described and classified taxonomically based on their elaborate cellular morphologies and behavior for over three centuries [1]. In the past decade, cultivation independent surveys of microbes have revolutionized our understanding of microbial diversity [2]. We now recognize that our reliance upon cultivation to identify and quantify microbes has resulted in missing upwards of 95% of extant bacterial and archaeal diversity [3]. Eukaryotic microbial diversity has received comparably less attention from sequence-based diversity surveys [4].

Recent eukaryote-specific cultivation-independent studies to assess the extent of microbial eukaryotic diversity have identified many novel taxa at a range of taxonomic levels – from novel species to novel phyla [5]–[9]. These surveys not only provide more comprehensive sequence data for inferences of phylogenetic relationships among diverse eukaryotes, but also provide *in situ* analyses of protists in natural environmental samples. It may seem astounding that we could be unaware of phylum-level protistan taxa [10]; however, the discovery of novel eukaryotic ssu rRNA genes in natural environmental samples mirrors the gaps in our understanding of bacterial and archaeal diversity. Virtually every time we have surveyed an environment using ssu rRNA cultivation-independent methods, we have found it contains more types of protists than we know from our morphological descriptions, culture collections or sequence databases. The current abundance of uncultivated eukaryotic sequence data confirms the incredible diversity of microbial eukaryotes in a variety of environments [11], [12]. The true extent of protistan diversity remains controversial; however, due to discrepancies with sequence-based identifications as compared to more traditional morphology-based descriptions of protistan diversity.

While ssu rRNA surveys provide information about eukaryotic phylotypes and the abundance of these types present in any given environment, there are few morphological descriptions that link a particular environmental ssu rRNA sequence to a specific morphological type. The appeal and ease of molecular community analyses has populated the databases with an abundance of sequence data from environmental samples in conjunction with little to no morphological data [13]. Despite the classic use of microscopy to identify and classify protists based solely upon morphology, purely structural descriptions of protists have limited applicability for modern assessments of microbial diversity, function, and community structure in natural environmental samples. Further, due to the complexity of life stages in some protists, even previously described protists can suffer from misclassification as distinct species in the absence of genetic data [1], [14]. Morphological features of protists may also be lost upon extended cultivation [15]. Thus a major challenge in describing true extant protistan diversity in diverse environments lies in connecting ssu rRNA sequence-based protistan diversity survey data with classical morphology-based descriptions.

The key ecological roles and importance of microbial eukaryotes in global geochemical cycling as either primary producers or consumers are also just being recognized. Eukaryotic specific sequence-based ssu rRNA surveys of eukaryotic diversity permit the *in situ* identification of protistan species based on phylotype [16]. Fluorescently labeled, ssu rRNA-targeted oligonucleotide probes are designed to hybridize to ssu rRNA sequences of protistan species or higher taxonomic clades. Such “phylogenetic stains” are used in fluorescent *in situ* hybridization (FISH) to visualize uncultivated protists, define their *in situ* spatial distribution, quantify their relative abundance within a natural environmental sample, and estimate their *in situ* physiological activity [17]. Microscopic examinations (light, fluorescence, electron) are, therefore, crucial to describe key morphological features of novel protists. A limitation of using whole cell rRNA-targeted FISH for the identification of microbial eukaryotes is that it does not provide morphological or structural information that could be corroborated with previously described protists that lack a sequenced ssu rRNA gene [18].

While there are a multitude of classical microscopic descriptions of protists, the skyrocketing number of uncultivated protistan sequences in our genetic databases lack corresponding morphological or physiological data [16]. To link ssu rRNA sequence data of uncultivated protists with traditional microscopic descriptions of protist morphology, we demonstrate here several methods for combining fluorescent *in situ* hybridization with both cytoskeletal or organellar immunostaining. Eukaryote-specific ssu rRNA-targeted immunoFISH can easily be used with commercial vital dyes for cytological markers such as Mitotracker® for staining mitochondria or phalloidin for staining actin. The method allows phylogenetic identification of an uncultivated protist using a whole cell rRNA-targeted FISH probe and immunostaining of informative cytological markers to be performed simultaneously on the same environmental sample. In contrast to other methods used to link sequence with morphology [19]–[21], immunoFISH is high throughput and permits detailed morphological descriptions without prior taxonomic knowledge. Lastly, this method can help to describe members of many of the novel protistan lineages reported in natural environmental samples.

## Results

### Whole cell rRNA-targeted FISH using the eukaryote ssu rRNA probe with microtubule cytoskeletal staining

**Figure 1:**
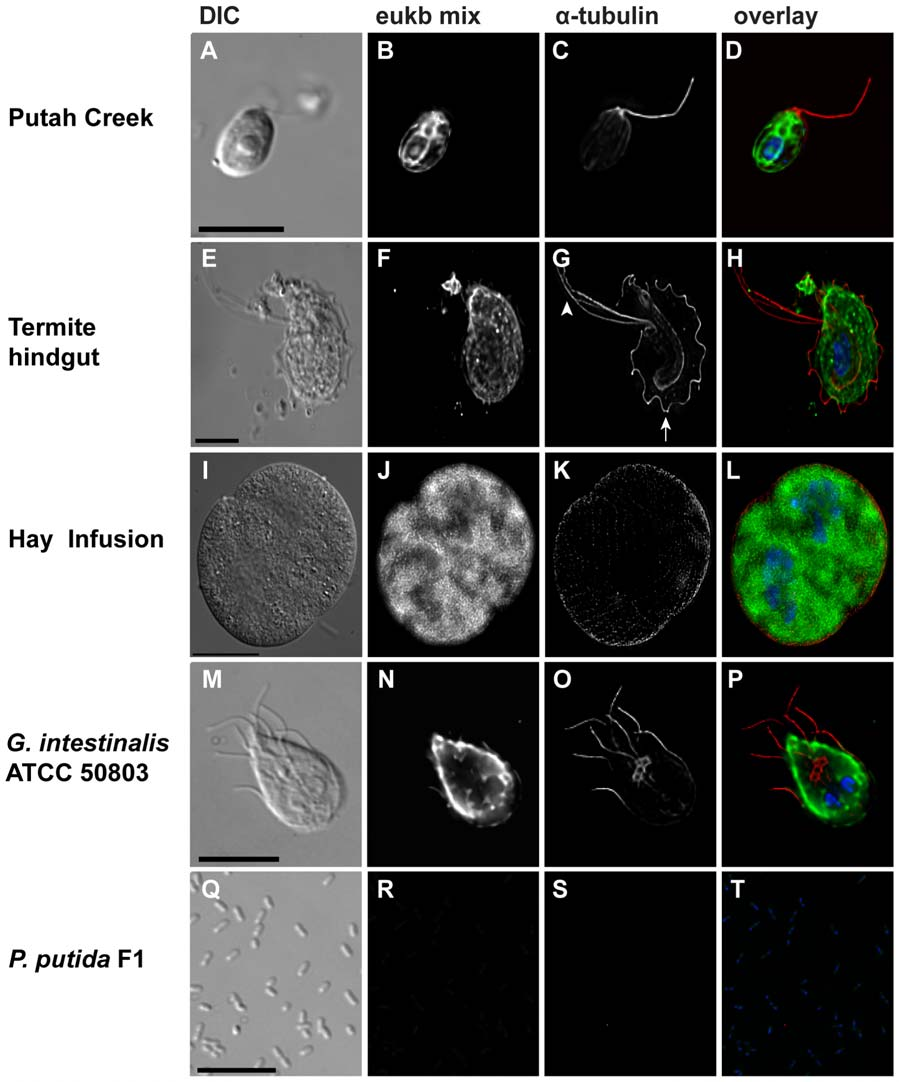
ImmunoFISH links phylotype with cytoskeletal morphology in protists from three environments. Representative immunoFISH of protists in Putah Creek (A–D), the termite hindgut (E–H), and the hay infusion enrichment (I–L) are presented. Positive control of the protist *G. intestinalis* ATCC 50803 (M–P) and negative control of the bacterium *P. putida* F1 (Q–T) are also shown. Fixed samples were hybridized with the broad eukaryote ssu rRNA probe (B, F, J, N, R) and overlaid with an anti-α-tubulin antibody to stain cytoskeletal features of each cell (C, G, K, O, S). The image overlays (also 3D stack in Video S1) show the eukaryote ssu rRNA probe (green), anti-α-tubulin antibody (red), and DAPI nucleic acid stain (blue) (D, H, L, P, T). Scale bars = 10 µm with the exception of the hay infusion enrichment (I) with the scale bar = 25 µm.

The eukaryotic ssu rRNA probe hybridized with all eukaryotes in the three environments tested after FISH protocol parameters were optimized (Figure 1). The eukaryote ssu rRNA probe hybridized with a variety of protists (e.g., diatoms, *Chlamydomona*s spp., and *Phacus* spp.) from the Putah Creek (Davis, CA) sample. The predominant protist in this environment was a small, ovular cell of approximately 6 µm (Figure 1 B). Combining the eukaryotic ssu rRNA probe in FISH with cytoskeletal immunostaining using the α-tubulin antibody, we observed one visible flagellum (the second was located under the body of the cell), as well as internal tubulin structure (Figure 1 C, D). This was indicative of a *Chlamydomona*s sp.

In the termite hindgut sample, the eukaryote ssu rRNA probe hybridized with previously identified protists such as *Trichomitopsis* (Figure 1 F), *Pseudotrichonympha*, *Dinenympha*, and *Streblomastix* as well as two to three smaller (1–3 µM) flagellates (data not shown). Using the α-tubulin antibody [22] with the eukaryote ssu rRNA FISH probe, we observed the complex flagellar structure of *Trichomitopsis*, including three extended flagella and one recurrent flagellum encapsulating the cell (Figure 1 G, H) [23].

Lastly, in the hay infusion sample, the eukaryote ssu rRNA probe hybridized with several types of ciliates, amoebae, and flagellates. One of the predominant protists from this environment was specifically, a *Colpoda* sp. in its various life stages, including during cell division (Figure 1 J). Using the eukaryote ssu rRNA probe in FISH with the α-tubulin antibody [22], we found that cilia were visible covering the external surface of the ciliate, and were easily seen during cell division (Figure 1 K, L). There was no visible internal microtubule structure.

The eukaryote ssu rRNA probe easily hybridized to the positive control *Giardia intestinalis* ATCC 50803, identifying the teardrop shape of the cell (Figure 1 N). Immunostaining with the α-tubulin antibody [22] revealed the four pairs of flagella (anterior, lateral, ventral, and posteriolateral on the cell body) as well as the median body structure unique to *Giardia* (Figure 1 O, P) [24]. Alternatively, the eukaryote ssu rRNA probe did not hybridize to bacterium *Pseudomonas putida* F1 (Figure 1 R), the negative control, so that its morphology was only visible in DIC (Figure 1 Q). The α-tubulin antibody [22] did not stain any tubulin structure, as *P. putida* F1 lacks a microtubule cytoskeleton (Figure 1 S, T).

### Whole cell rRNA-targeted FISH using the ciliate-specific ssu rRNA probe with microtubule cytoskeletal staining

**Figure 2:**
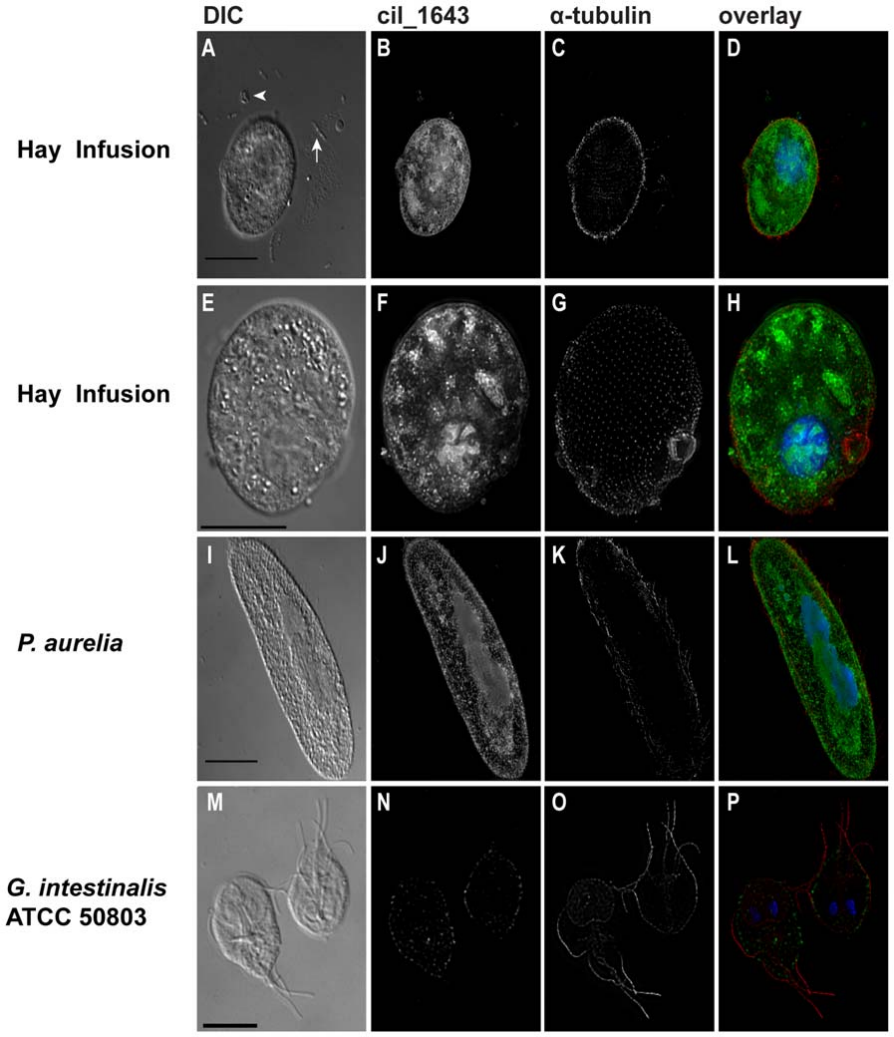
ImmunoFISH links ciliate-specific phylotypes with their cytoskeletal morphologies in the hay infusion enrichment. Ciliate positive control *P. aurelia* (I–L) and the diplomonad negative control *G. intestinali*s ATCC 50803 (M–P) are also shown. Fixed samples were hybridized with the ciliate-specific ssu rRNA probe (B, F, J, N) and overlaid with an anti-α-tubulin antibody to stain cytoskeletal features of each cell (C, G, K, O). The image overlays (also see 3D stack in Video S2) show the ciliate-specific ssu rRNA probe (green), anti-α-tubulin antibody (red), and DAPI nucleic acid stain (blue) (D, H, L, P). The tailed arrow marks a bacterium, and the arrowhead marks an amoeba. Scale bars = 25 µm with the exception of the positive control *G. intestinalis* ATCC 50803 (M) with the scale bar = 10 µm.

We developed and optimized a ciliate-specific ssu rRNA FISH probe that hybridized only with ciliates (not amoebae, flagellates, or bacteria) in the hay infusion environment under optimized FISH parameters (Figure 2 A–D). The combination of the ciliate-specific ssu rRNA probe and the α-tubulin antibody [22] immunostaining revealed cilia present over the entire cell body, reminiscent of the combination of the eukaryote ssu rRNA probe with the α-tubulin antibody [22] (Figure 2 F–H). As a positive control, we demonstrated that the ciliate-specific probe hybridized to the ciliate *Paramecium aurelia* (Figure 2 J), and the anti-α-tubulin antibody revealed cilia covering the cell body (Figure 2 K, L). As expected, the ciliate-specific ssu rRNA probe did not hybridize to *G. intestinalis* ATCC 50803, a flagellated diplomonad protist (Figure 2 N); however, immunostaining with the α-tubulin antibody [22] marked the eight *Giardia* flagella (Figure 2 O, P).

### Whole cell rRNA-targeted FISH using the eukaryote ssu rRNA probe with actin cytoskeletal staining

**Figure 3:**
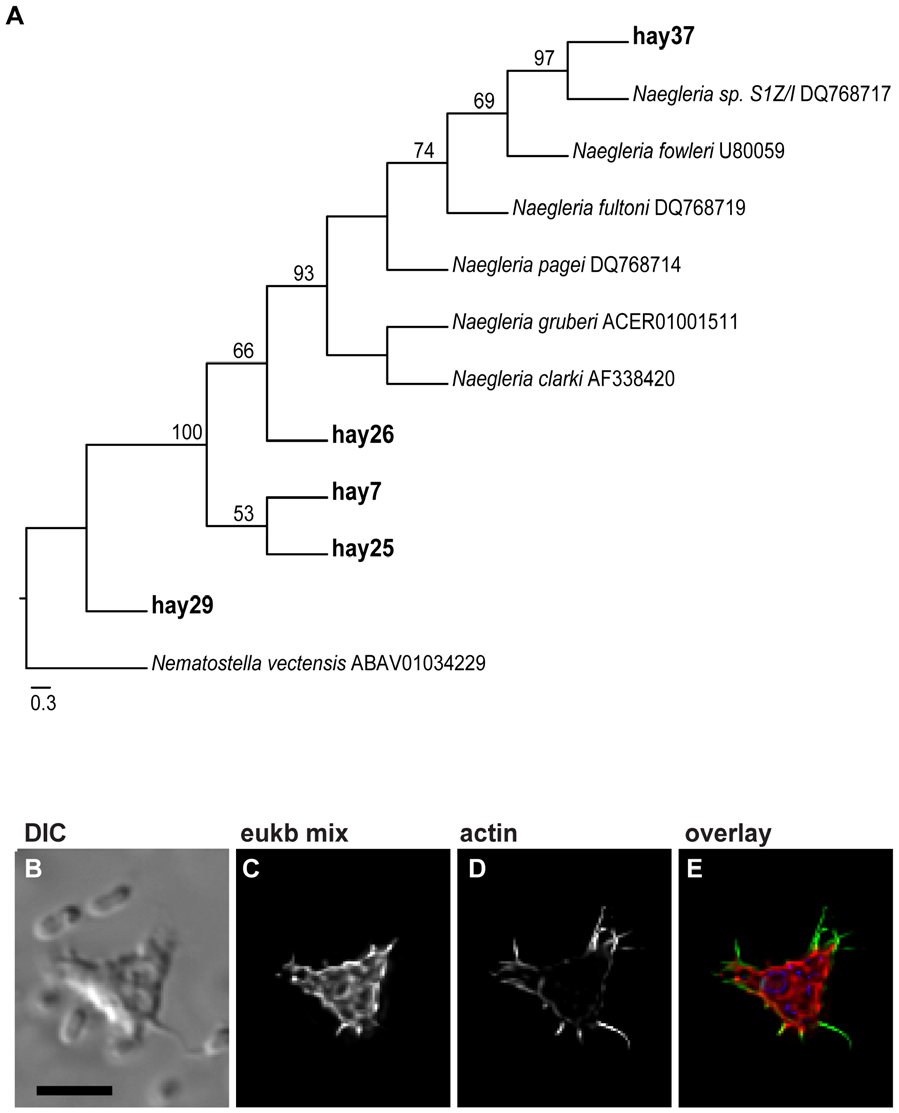
Amoebae found in the hay infusion enrichment are closely related to *Naegleria* spp. The evolutionary relationships of the rDNA sequences from the hay infusion were determined by bootstrap analysis using RAxML and are presented in A (only bootstrap values ≥50% are shown above the branches). Accession numbers follow the species name and sequences identified in this study are represented by the name “hay” followed by the accession number (A). The eukaryotic rRNA-targeted FISH (C) overlaid with the phalloidin (actin) stain (D) links phylotype with amoeboflagellate morphology in the hay infusion enrichment (B–E). The image overlay (also see 3D stack in Video S3) shows the eukaryote ssu rRNA probe (green), phalloidin stain (red), and DAPI nucleic acid stain (blue) (E). Scale bar = 10 µm.

To link morphological descriptions of amoebae with rRNA-based phylogenetic information, we added fluorescently labelled phalloidin, an actin-binding stain, to the hybridization buffer during FISH. The eukaryote ssu rRNA probe hybridized with all eukaryotes in the hay infusion (Figure 1 J, Figure 3 C). In conjunction with the phalloidin stain, amoebae were specifically visible due to the staining of the actin cytoskeleton (Figure 3 D, E). The prominent actin cytoskeleton in the amoebae was particularly notable when the amoebae extended their pseudopodia (Figure 3 D, E).

### Staining of mitochondria and hydrogenosomes combined with whole cell rRNA-targeted FISH using the eukaryote ssu rRNA probe

**Figure 4:**
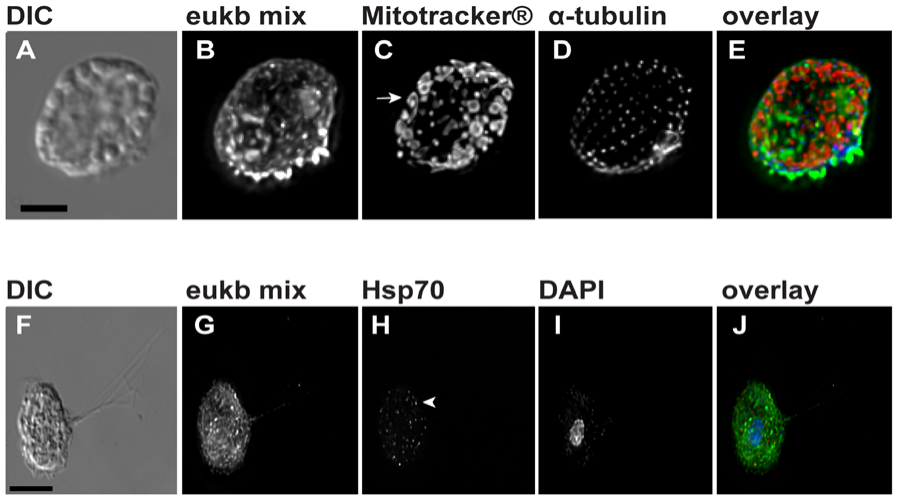
ImmunoFISH in two environments links eukaryotic phylotypes with the subcellular localization of their mitochondria or hydrogenosomes. Live hay infusion (A–E) was incubated with Mitotracker® Red CM-H_2_XRos (C), fixed, hybridized with the eukaryote ssu rRNA probe (B), and overlaid with an anti-α-tubulin antibody to stain cytoskeletal features of each cell (D). Tailed arrow marks ovoid mitochondria. The image overlay (E) shows the eukaryotic ssu rRNA probe (green), Mitotracker® Red CM-H_2_XRos (red), and anti-α-tubulin (blue). Scale bar = 10 µm. Fixed termite hindgut samples (F–J) were hybridized with the eukaryote ssu rRNA probe (G), overlaid with and anti-Hsp70 antibody to stain hydrogenosomes (H, arrowhead). The image overlay (J) shows the eukaryotic ssu rRNA probe (green), anti-Hsp70 antibody (red), and DAPI nucleic acid stain (blue). Scale bar = 25 µm. Also see 3D stacks of overlays in Video S4.

As described previously, we found that the eukaryote ssu rRNA probe hybridized with all eukaryotes in the hay infusion including smaller ciliates (Figure 1 J, Figure 3 C, Figure 4 B, E). Cilia were visible on the surface of the cell in an organized spatial arrangement by anti-α-tubulin immunostaining (Figure 4 D, E). To define the subcellular localization of mitochondria in the ciliates, we used the vital dye Mitotracker®. In the same cells, we observed ovoid mitochondria localized throughout the cytoplasm of the cell (Figure 4 C, E).

The termite hindgut contains many types of anaerobic protists that lack mitochondria, but in some instances possess another energy generating organelle termed the hydrogenosome [25]. When we combined the eukaryote ssu rRNA probe (Figure 4 G, J) with an anti-hsp70 antibody specific to hydrogenosomes, we observed hydrogenosomes in the cytoplasm of *Trichomitopsis* (Figure 4 H, J) as well as some of the other flagellates in the termite hindgut (data not shown).

### Protistan diversity in the hay infusion enrichment

To determine the protistan diversity of the hay infusion enrichment, we sequenced eukaryotic ssu rDNA clones in both directions. These sequences were aligned using secondary structure-based alignment in ARB [26] and the evolutionary relationships of eighteen unique sequences determined by phylogenetic analysis using RAxML (Figure 3 A, Figure S1) [27]. Four of these sequences grouped significantly and closely with many species of the amoeboflagellate *Naegleria* (Figure 3 A). The sequence hay37 is likely a different, but related, species of the amoeboflagellate *Naegleria* sp. S1Z/I, because it has strong bootstrap support for a separate branch within the amoeboflagellate clade. Sequences hay26, hay7, and hay25 form a polytomy within this clade suggesting their placement within the amoeboflagellate clade is unclear. The clone hay29 is not a part of the amoeboflagellate clade; however, when this sequence was analyzed in BLAST, a high e-value (0) and sequence similarity (99.87%) identify this sequence as most closely related to *Naegleria* sp. F1-28.

In addition to the presence of amoeboflagellates in the hay infusion enrichment, we found rDNA sequence phylotypes that grouped with known ciliates, primarily *Colpoda* and related genera (Figure S1). This confirms immunoFISH results obtained using the ciliate-specific ssu rRNA probe with the anti-α-tubulin antibody [22] that identified *Colpoda*-like ciliates (Figure 2). Several of these sequences formed a strongly supported ciliate clade, but a few strongly supported sequences did not fall within the ciliate clade (Figure S1). Secondly, three sequences fell into two well-supported clades of other flagellated protists including the Fungi and the Cercozoa (Figure S1).

## Discussion

Several methods to link ssu rRNA sequence data with the morphology of protists have been proposed including the use of FISH with silver stain techniques, scanning electron microscopy (SEM), and single cell approaches [19]–[21]. Combining FISH with silver staining techniques facilitates the quantification of targeted fixed cells in an environmental sample, but provides limited information about morphological features. FISH in combination with silver staining can identify certain morphological features in ciliates, because the oligonucleotides used in FISH solely target rRNA (ribosomes) and therefore the macro- and micronucleus are visible as well as vacuoles and the vestibulum [19]. For the most part, however, silver staining allows for easier quantification of cells hybridized to a specific probe in FISH and does not provide exhaustive morphological descriptions [19]. Alternatively, combining FISH with SEM allows for high-resolution visualization and easy detection of key morphological characteristics across many protistan phylogenetic groups. For example FISH combined with SEM revealed the classical tabulation pattern and clear ornamentation of thecal plates of the dinoflagellate *Peridinium cinctum* [20]. Unfortunately, the combined use of FISH and SEM does not lend itself well to high-throughput analysis of microbial eukaryotes in a community. While SEM can be used to assess external structural components of protists, internal structures that may be crucial in identifying a particular taxonomic group are largely ignored.

Parallel molecular and morphological techniques have been applied to individual protistan cells using single-cell extraction techniques, ssu rRNA sequencing coupled with microscopic descriptions of morphology from live cells, and confirmation of structure with SEM. Duff *et al.* (2008) examined 12 major groups of planktonic protists using this approach and were able to provide parallel morphological descriptions of each group along with their phylogenetic identities using ssu rRNA data [21]. However, initial taxonomic identification using light microscopy is a crucial step for this method. Secondly, DNA contamination is a possibility, which was reflected in a mismatch between described morphological features and the ssu rRNA sequence identification from the same sample. The protist cells from the Duff *et al.* (2008) study were also large and conspicuous, and smaller cells may be difficult to isolate as single cells [21]. This “single cell” approach to link sequence and morphology of protists is powerful; however, a more streamlined, less labor-intensive, high throughput method may allow broader descriptions of microbial eukaryotes within an entire community.

The approach described here will help to rectify these issues. Samples from three environments – a freshwater creek, a hay infusion enrichment, and the termite hindgut – confirm the utility of the immunoFISH method for linking phylogenetic sequence with morphology. Samples can be taken directly from the environment, fixed, and attached to slides or coverslips for microscopic analysis. While the use of ssu rRNA-targeted probes with FISH can identify individual microbial eukaryote cells in any given environment, the use of FISH alone provides only the general shape and size of any given cell (Figure 1 B, F, J, N). Information about cytoskeletal structure or internal features of the eukaryotic cell is obtained using antibodies and dyes to stain relevant cytological markers. For high throughput protistan diversity surveys that provide only ssu rDNA sequence, immunoFISH with cytoskeletal markers can readily link ssu rDNA phylotypes with key morphological features of novel species, without prior morphological taxonomic knowledge.

### Linking ssu rRNA phylotypes with microtubule immunostaining to describe flagellate and ciliate morphology

Microtubule immunostaining is particularly informative for describing the morphology of flagellates and ciliates. Combining whole cell ssu rRNA-targeted FISH with microtubule immunostaining provides information about the number and location of flagella on the cell, as well as any internal microtubule structures. In environments in which ssu rRNA sequence data is exclusively available, screening initially with a broad eukaryotic probe and an anti-α-tubulin antibody can provide basic descriptions of all eukaryotes within that particular environment. This initial screen can then be followed up with immunoFISH using more specific probes to particular taxonomic groups (e.g., genera or species) and an optimized antibody to a specific cellular organelle or structure.

We have shown the utility of the immunoFISH method by broadly targeting flagellates and ciliates using a eukaryote ssu rRNA probe in three different environments (Figure 1) as well as a ciliate-specific ssu rRNA probe in the hay infusion enrichment (Figure 2). The hay infusion contained an abundance of different types of protists including flagellates, amoebae, and fungi (Figure 3 A, Figure S1). The majority of the diversity was found in the Family *Ciliophora*, thus we designed and optimized a ciliate-specific ssu rRNA probe. The ciliate-specific ssu rRNA probe was hybridized with the hay infusion sample in conjunction with the anti-α-tubulin antibody to exclusively target ciliates. The ciliate-specific probe enabled identification of different types of ciliates within the hay infusion, including *Colpoda* spp. and *Pseudoplatyophrya* spp. Anti-tubulin immunostaining also revealed the presence of regularly spaced surface cilia on the cell body of an abundant morphological type (likely a *Colpoda* sp.), and also revealed the location of the oral apparatus surrounded by microtubule ribbons in one hay infusion ciliate (Figure 2, E–H). The use of the ciliate-specific probe, therefore, allowed for the identification of a *Colpoda* sp., which was also one of the most common uncultivated rRNA sequences in the correlating clone library from the hay infusion (Figure S1).

### Linking ssu rRNA phylotype with actin staining to describe amoeboid morphology

Using live imaging, we observed that the hay infusion pellicle also contained numerous small amoebae. We classified four ssu rDNA sequences from the pellicle as belonging to the amoeboflagellate clade composed of exclusively *Naegleria* spp. with strong bootstrap support (Figure 3 A). The evolutionary relationships of the rDNA clones hay7, hay25, hay26, and hay29 are less certain, although this clade likely represent unique species-level diversity. *Naegleria* spp. can exist in the environment as amoebae or transform into other morphological types such as flagellates or cysts.

Small amoebae are abundant in many aquatic environments [28], [29], but can often be difficult to identify based solely upon morphology due to a lack of obvious morphological characters. Amoebae are more often characterized based on their type of movement [28], [29], and many protists have complex life cycles like *Naegleria* spp. that include amoeboid and flagellate stages. Because amoeboid lineages occur within many eukaryotic groups, it is often difficult to infer the identity of an amoeboid protist based solely upon ssu rRNA sequence. Amoebae cytoskeletons are primarily composed of actin, thus we combined a common actin stain (phalloidin) in whole cell rRNA-targeted hybridization to link phylogenetic sequence with a morphological description of the amoeba in the hay infusion enrichment (Figure 3 B–E). Using our ssu rRNA sequence data, we were able to identify several amoeboflagellates grouping with the *Naegleria* spp. clade (Figure 3 A). We were then able to confirm amoeboid morphology and link it to phylogenetic sequence data using the eukaryote ssu rRNA probe along with a common actin stain as a proof of principle. An advantage of a using a stain like phalloidin rather than an antibody is that antibody optimization and titration are not needed.

We also identified putatively non-amoeboid sequences in the hay infusion enrichment ssu rDNA library. These included several sequences affiliated with ciliates, fungi, or cercozoans (Figure S1). Several sequences (hay14, hay21, hay31 and hay20) had no specific affiliation with known groups, as supported by a less than 97% sequence similarity (Figure S1). The protists represented by these unique sequences could easily be targeted using specific ssu rRNA FISH probes combined with actin or tubulin staining.

### Subcellular localization of mitochondria and hydrogenosomes in uncultivated protists

Another cytological feature of both aerobic and anaerobic microbial eukaryotes is the presence of energy generating organelles such as mitochondria or hydrogenosomes. We used two strategies, immunostaining and vital dyes, to mark mitochondria and hydrogenosomes in uncultivated protists along with whole cell ssu rRNA-targeted FISH. Because mitochondrial morphology can be used as a key feature to classify protists [1], we used Mitotracker® in conjunction with immunoFISH (using anti-α-tubulin) to characterize the morphology and abundance of mitochondria in uncultivated ciliates, flagellates, and amoebae. In the diverse hay infusion enrichment, visible ovoid mitochondria were present throughout the cytoplasm of some ciliate cells (Figure 4 A–E), while other protists contained visible circular mitochondria but lacked tubulin cytoskeletal structure (not shown).

Anaerobic protists lack mitochondria, but may possess other energy-deriving organelles such as hydrogenosomes [25]. To target hydrogenosomes in eukaryotes within the termite hindgut, we used a hydrogenosomal-specific anti-hsp70 polyclonal antibody in conjunction with the eukaryote ssu rRNA probe (Figure 4 F–J). The conserved nature of hydrogenosomal Hsp70 allowed for sufficient cross-reactivity of the anti-Hsp70 polyclonal antibody to Hsp70 within termite hindgut flagellates [30]. Although the hydrogenosomal-specific anti-Hsp70 antibody is polyclonal, it may not target hydrogenosomes in all anaerobic protists because epitope sites may vary. As an alternative to using an antibody to target hydrogenosomes, the commercial probe Mitotracker® could also be used as described above for aerobic protists.

### Optimization of whole cell ssu rRNA-targeted FISH combined with staining of cytological markers

Small subunit (ssu) rRNA-targeted whole cell FISH requires that intact rRNA be present and in high abundance in a cell in order for an rRNA-targeted FISH probe to bind properly and produce a positive signal. Thus, using freshly fixed cells (within 2 weeks) will increase the probability that rRNA will be undamaged and useful for obtaining a positive signal in FISH. Secondly, for each new environment and probe, the stringency conditions (temperature, formamide concentration, type of detergent) for FISH should first be optimized with candidate fluorescently labeled probes to ensure that the intended protists are targeted, and to minimize non-specific hybridizations. Lastly, positive and negative controls for the candidate FISH probes are imperative for validating whole cell ssu rRNA-targeted FISH experiments [31]. This is obviously more difficult with previously undescribed eukaryotic microbes, particularly those that may exist in several different life stages with different morphologies. The identification by sequence and subsequent morphological characterization of novel protists found in disparate environments, however, should lend to a consensus description of novel uncultivated protistan taxa.

The immunostaining portion of immunoFISH should also be optimized. The antibody of choice must be tested to ensure that it binds to the given protein within the targeted eukaryotic cells, as conserved antibodies targeting specific taxonomic groups of microbial eukaryotes may not bind to epitope sites on all protists. Secondly, it is essential to have positive and negative controls for the antibody to confirm the results of the immunostaining portion of the method. Lastly, antibodies should be qualitatively titrated to ensure that the minimum amount of antibody is used to detect a signal (both the primary and secondary antibody) and to keep the signal above the background for imaging.

Commercial stains such as Mitotracker® and phalloidin often have guidelines for establishing signal within targeted cells, but these protocols are often designed for eukaryotic cell lines and not uncultivated microbial eukaryotes. Optimizing the use of the commercial probes in conjunction with immunoFISH may also be necessary as described (see Methods).

Cultivation-independent molecular approaches to identify protists by ssu rRNA sequences allow us to map the true diversity and evolutionary relationships of microbial eukaryotes in the natural world. When linked to phylogenetic tags such as ssu rRNA, microscopic descriptions can complement and help to vet the increasing numbers of uncultivated protists identified in diverse environments. The immunoFISH method provides a fast, efficient method for linking phylogenetic sequence with morphology for microbial eukaryotes in any natural environment, especially novel uncultivated protists lacking any morphological information. There are several benefits in the use of immunoFISH over other methods such as FISH combined with SEM or single-cell sequencing with microscopy. The immunoFISH method requires only a small sample and no prior knowledge of protist taxonomy, and can be used to describe ubiquitous as well as rare protists of various sizes. Phylogenetically targeted whole cell ssu rRNA-targeted FISH of protists, bacteria and/or archaea, when combined with cytological staining, can demonstrate the subcellular localization of endosymbionts and organelles respectively. This approach could also be expanded to include other stains for eukaryotic structures, such as LysoSensor (Invitrogen) for lysosomes.

Next generation sequencing approaches to sample the *in situ* diversity of protists have revealed unprecedented numbers of novel protistan taxa [32]. Claims of novel protistan diversity, however, will ultimately need to be confirmed using microscopy and metagenomic investigations. Shorter pyrosequencing reads permit us to identify and classify large numbers of environmental protists [33], but these shorter sequence reads must either be mapped onto full length sequences for accurate phylogenetic identification, or used as “phylogenetic stains” in rRNA-targeted fluorescent *in situ* hybridizations to identify target organisms [31] as described here. ImmunoFISH can also be high-throughput as it requires only minimal optimization for each new environment, FISH probe and/or antibody. The application of this method, as well as other molecular genetic strategies, will help us to understand the true nature and extent of protistan diversity.

## Methods

### Sample collection

Water samples were collected from Putah Creek at 38°31′48.65″ N and 121°45′35.46″ W on the University of California, Davis campus. The water samples were filtered through 0.22 µm filters to concentrate protists. A hay infusion enrichment containing protists was prepared by adding 500 ml of tap water to a sterile Erlenmeyer flask containing oat hay and alfalfa. The flask was covered and incubated in the light for 6–25 days. *Zootermopsis angusticollis* termites (Ward's™ Natural Science) were frozen at −80°C. The head of the termite was excised with a sterile razor blade, and forceps were used to remove the digestive tract from the body. The hindgut was punctured and 100 µl of 1X HEPES-buffered saline, pH 7.2 (1.0 g/L dextrose, 5 g/L HEPES, 0.37 g/L KCl, 8 g/L NaCl, 0.135 g/L Na_2_HPO_4_-H_2_O) was used to suspend the contents.

### Sample fixation

Concentrated protists from Putah Creek, hay infusion protists, and resuspended hindgut contents were fixed in 4% paraformaldehyde, and incubated at room temperature for 20–30 min. Fixation was quenched by washing twice in PEM buffer (100 mM PIPES, 1 mM EGTA, 0.1 mM MgSO_4_), followed by low speed centrifugation (500–900*× g*). The supernatant was removed, the cells suspended in PEM buffer, and stored at 4°C.

### Cytoskeletal and organellar staining combined with whole cell rRNA-targeted FISH

Slides were coated with poly-L-lysine to promote attachment of fixed cells from each of the environmental samples or enrichments. Samples were incubated on the slides at room temperature for 10–20 minutes and slides were incubated with pre-warmed hybridization buffer (900 mM NaCl, 20 mM Tris-HCl, pH = 8, 0.01% detergent, and, sometimes, formamide for specificity [see Table 1]) in a 46°C water bath in a humidifying chamber. One of two ssu rRNA FISH probes were used for hybridization, a general eukaryote probe or a ciliate specific probe (5′- CACTCGRAATCGGTAGRAGCG -3′), cil_1643 (numbering based on *Saccharomyces cerevisiae* rRNA structure). The general eukaryote probe (eukb mix) was composed of three probes used simultaneously, E309 (5′- TCAGGCBCCYTCTCCG -3′), E503 (5′- GGCACCAGACTKGYCCTC -3′), and E1193 (5′- GGGCATMACDGACCTGTT -3′) [34].

**Table 1:** Specific conditions for the immunoFISH protocol for each environment or control sample.

Environment	FISH Hybridization Conditions					FISH Wash Conditions		Immunostaining Conditions	
	Probe	Conc (ng/µl)	Detergent	Formamide	Time (h)	NaCl (mM)	Temp (°C)	Primary Ab^4^	Secondary Ab
Putah Creek Davis, CA	eukb mix^2^	3.0 ea	Triton-X	25%	16	149^3^	48	TAT-1 (1∶200)	Alexa®594 (1∶400)
Termite Hindgut (α-tubulin)	eukb mix^2^	3.0 ea	SDS	10%	3	450	46	TAT-1 (1∶500)	Alexa®594 (1∶500)
Termite Hindgut (Hsp70)^1^	eukb mix^2^	3.0 ea	SDS	10%	3	450	46	TAT-1 (1∶5000)	Alexa®555 (1∶5000)
Hay Infusion	eukb mix^2^	2.5 ea	SDS	10%	3	450	46	TAT-1 (1∶500)	Alexa®594 (1∶500)
*Giardia* ATCC 50803	eukb mix^2^	2.5 ea	Triton-X	10%	16	450	48	TAT-1 (1∶500)	Alexa®594 (1∶500)
*P. putida* F1	eukb mix^2^	2.5 ea	Triton-X	10%	16	450	48	TAT-1 (1∶500)	Alexa®594 (1∶500)
Hay Infusion	cil-1643	5.0	SDS	0%	3	900	46	TAT-1 (1∶500)	Alexa®594 (1∶500)
*P. aurelia*	cil-1643	5.0	SDS	0%	3	900	46	TAT-1 (1∶500)	Alexa®594 (1∶500)
*Giardia* ATCC 50803	cil-1643	5.0	SDS	10%	3	900	46	TAT-1 (1∶500)	Alexa®594 (1∶500)

1 *Trichomonas vaginalis* anti-Hsp70 antibody.

2 The eukb mix is composed of three probes used simultaneously.

3 For Putah Creek, 5 mM EDTA was added to the wash buffer.

4 Antibody dilution is shown in parentheses.

Following hybridization, slides were washed twice with pre-warmed wash buffer (from 149–900 mM NaCl, 20 mM Tris-HCl, pH = 8, 0.01% detergent) in a heated water bath (Table 1) for 20 minutes. Slides were then blocked in PEMBALG (1 M PIPES, 500 mM EGTA, 100 mM MgSO_4_, 1% bovine serum albumin, 0.1% NaN_3_, 100 mM L-lysine monohydrochloride, 0.5% cold water fish skin gelatin) at room temperature for 45 minutes. The PEMBALG was then aspirated and a primary antibody (50 ul), diluted in PEMBALG (Table 1), was added to each slide and incubated at room temperature for 45 minutes. Following incubation, slides were washed three times with PEMBALG. A secondary antibody (50 ul), diluted in PEMBALG (Table 1), was added to each slide, and the slides were again incubated at room temperature for 45 minutes. After incubation with the secondary antibody, slides were washed three times with PEMBALG followed by an additional three washes with PEM. Coverslips were mounted over the slides using 20 µl of Prolong® Gold Antifade reagent with DAPI (Invitrogen). Slides were stored in the dark for at least 20 hours before visualizing.

Image stacks were collected using a Leica DMI6000 B inverted fluorescence microscope with Differential Interference Contrast (DIC). Serial sections were acquired at 0.2 µm intervals. Data stacks were deconvolved using Huygens Deconvolution Software and two-dimensional projections were created from the three-dimensional data sets using ImageJ.

### Actin staining combined with whole cell rRNA-targeted FISH using the eukaryote ssu rRNA probe

Cells in 100 µl of live hay infusion were attached to poly-L-lysine coated coverslips for 10 minutes. After 10 minutes, the excess liquid was aspirated and cells were fixed in 3.7% paraformaldehyde in 1X HBS (pH = 7.2) for 10 minutes at room temperature. The excess liquid was aspirated and the coverslips were washed twice with 1X HBS. After aspiration, cells were hybridized with the eukb probe mix [34], (labeled with a Cy3 fluorophore). To stain the actin cytoskeleton, Alexa Fluor® 488 phalloidin (0.6 units, Invitrogen) was added concomitantly with the hybridization solution and eukb probe mix. Coverslips were hybridized in a water bath at 46°C for 3 hours and subsequently washed twice with wash buffer without detergent for 20 minutes at 46°C. Coverslips were mounted using 20 µl of Prolong® Gold Antifade reagent with DAPI.

### Mitochondrial staining using Mitotracker® Red CM-H_2_XRos combined with whole cell rRNA-targeted FISH using the eukaryote ssu rRNA probe

Live hay infusion (1 ml) was centrifuged at 900*× g* for 5 minutes at room temperature. The supernatant was aspirated and cells were resuspended in 1 ml of 1X HBS, pH = 7.2 with 1 µM Mitotracker® Red CM-H_2_XRos and kept at room temperature for 45 minutes. The cells were centrifuged at 900*× g* for 5 minutes, washed twice in 1X HBS, and fixed in 3.7% paraformaldehyde in 1X HBS for 15 minutes at room temperature. The cells were centrifuged again at 900*× g* for 5 minutes, washed twice with PEM, and resuspended in 1 ml of PEM. One hundred microliters of suspended cells were then attached to poly-L-lysine slides for 10 minutes. After 10 minutes, the excess liquid was aspirated and immunostaining was performed as above, although the secondary antibody used was AlexaFluor 350® donkey anti-mouse IgG and slides were mounted in 20 µl of Prolong® Gold Antifade reagent without DAPI.

### Sequence-based surveys of protistan diversity in the hay infusion enrichment

Approximately 1 ml of the pellicle of the hay infusion was sampled eight days after the initiation of the enrichment. This sample was centrifuged at 500*× g* for 5 minutes at room temperature. The supernatant was then decanted and the pellet frozen at −20°C. Total community genomic DNA was extracted from the pellet using a bead-beating protocol for soil DNA extraction [35] with the following modifications: only 2× Buffer A and lysozyme (5 mg/ml) were added to the sample, then the sample was mixed by inversion, and incubated while rotating at 37°C for 30 minutes. The final DNA pellet was air-dried and resuspended in nuclease free water. Total genomic DNA concentration was measured using the Nanodrop® ND-1000 UV-Vis Spectrophotometer.

### PCR amplification, cloning and sequencing

A fragment of the eukaryotic ssu rRNA gene was amplified using 246 pg of hay infusion DNA with the following master mix: 1X LA Taq Buffer, 0.2 mM dNTPs, 0.2 µM 360FE forward primer (5′- CGGAGARGGMGCMTGAGA -3′), 0.2 µM 1391RE reverse primer (5′- GGGCGGTGTGTACAARGRG -3′) [5], and 0.025 units of LATaq™ (TaKaRa). The following amplification profile was used: 95°C 5 minutes; 94°C 1 minute, 58.8°C 1 minute, 72°C 1 minute for 30 cycles; 72°C 10 minutes. The PCR amplicons were cloned directly into the PCR®2.1-TOPO® vector (Invitrogen). Forty-eight colonies were grown up in 100 µl of Luria Broth with 50 µg/µl kanamycin overnight at 37°C. Twenty microliters of 10 mM Tris pH = 8 was added to 20 µl of cells and boiled at 99°C for 10 minutes. Three microliters of the boiled cell suspension was added to a master mix composed of the following: 1X AmpliTaq Buffer, 2.5 mM MgCl_2_, 0.2 mM dNTPs, 0.04 mM T7 promoter primer, 0.04 mM M13 reverse primer, and 2.5 units of AmpliTaq Gold® DNA Polymerase (Invitrogen). PCR products were separated on a 1% agarose gel and visualized using ethidium bromide. Samples containing a properly sized insert were treated with ExoSAP-IT (Affymetrix-USB). Ten microliters of PCR product was treated with 1 µl of ExoSAP-IT and heated at 37°C for 45 minutes followed by heat inactivation at 80°C for 15 minutes. Samples were sequenced in two directions using Sanger sequencing (University of California, Berkeley DNA Sequencing Facility). Sequences were then added to the Silva Release 106 SSU ARB database and aligned [26]. The alignment was used to build maximum likelihood phylogenetic trees using RAxML utilizing the GTR-GAMMA substitution method with 1000 bootstrap runs [27].

## Supporting Information

Figure S1 **Maximum likelihood phylogenetic analysis of eukaryotic diversity in the hay infusion enrichment.** RAxML phylogenetic analyses indicate that the eukaryotic ssu rDNA sequences from the hay infusion library group into three clades: Ciliates, Fungi, and Cercozoans. Bootstrap values ≥50% are shown above the branches. Sequences identified in this study are represented by the name “hay” followed by the accession number.
(TIF)

Video S1 **Three dimensional deconvolved stacks from images in** **Figure 1 D, H, L, and P** **.**
(M4V)

Video S2 **Three dimensional deconvolved stacks from images in** **Figure 2 H, L, and P** **.**
(M4V)

Video S3 **Three dimensional deconvolved stacks from the image in** **Figure 3 E** **.**
(M4V)

Video S4 **Three dimensional deconvolved stacks from images in** **Figure 4 E and J** **.**
(M4V)
