# Supplementary figures and images for: Uncultivated Microbial Eukaryotic Diversity: A Method to Link ssu rRNA Gene Sequences with Morphology
Source: PLoS One. 2011 Dec 8;6(12):e28158. doi: 10.1371/journal.pone.0028158 (PMC3234254; doi:10.1371/journal.pone.0028158)

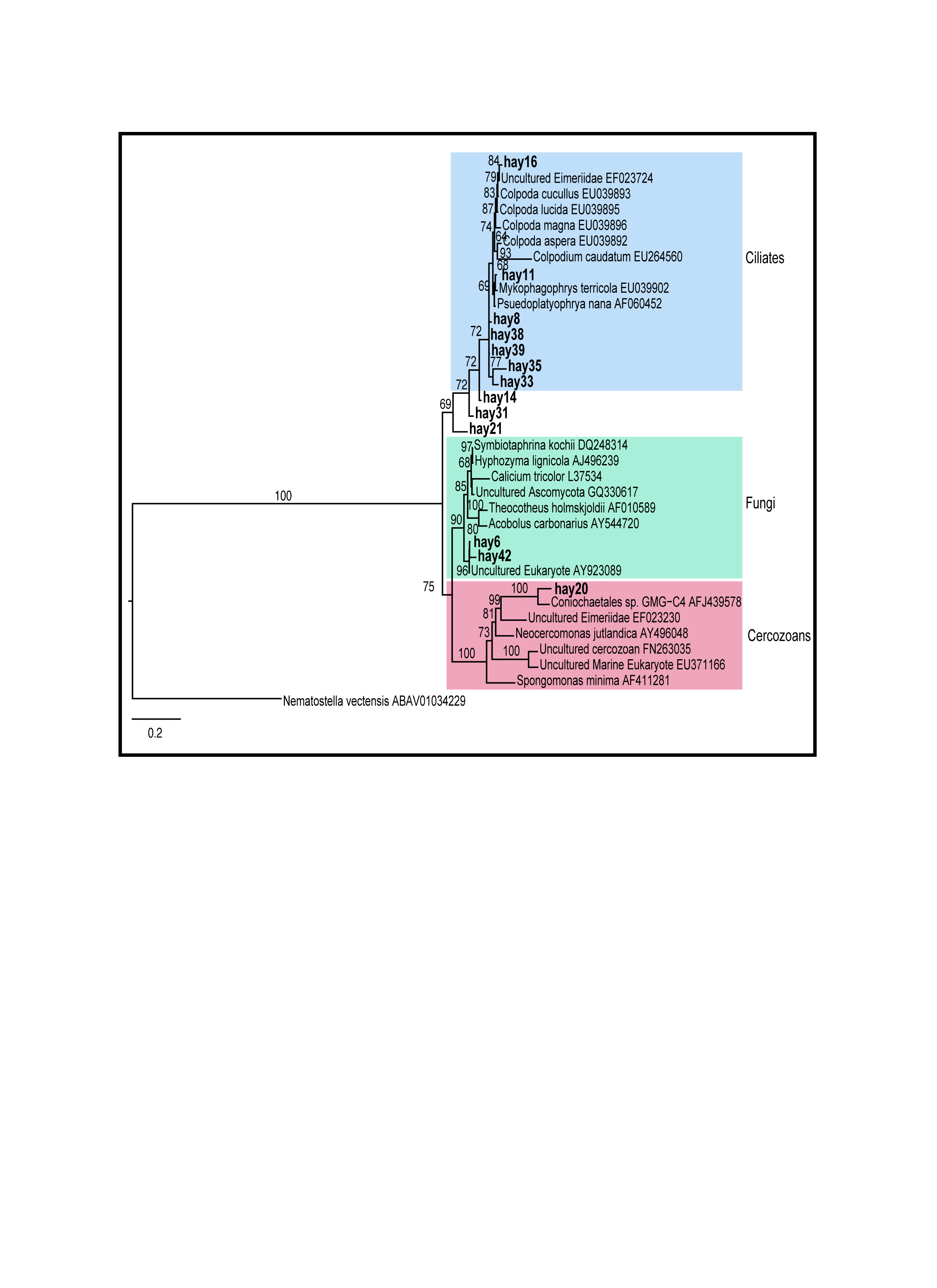

Supplement: Figure S1 — Maximum likelihood phylogenetic analysis of eukaryotic diversity in the hay infusion enrichment. RAxML phylogenetic analyses indicate that the eukaryotic ssu rDNA sequences from the hay infusion library group into three clades: Ciliates, Fungi, and Cercozoans. Bootstrap values ≥50% are shown above the branches. Sequences identified in this study are represented by the name “hay” followed by the accession number. (TIF) [file pone.0028158.s001.tif]
